# Executive Function and Contingency Management in Methamphetamine Use Disorder

**Published:** 2021-05-12

**Authors:** Lara J van Nunen, Marilyn T Lake, Jonathan C Ipser, Dan J Stein, Steven J Shoptaw, Edythe D London

**Affiliations:** 1 Department of Psychiatry and Mental Health, University of Cape Town, Neuroscience Institute, South Africa; 2 Department of Paediatrics and Child Health, University of Cape Town, Neuroscience Institute, South Africa; 3 South African Medical Research Council Unit on Risk & Resilience in Mental Disorders; 4 Department of Psychiatry and Neuroscience Institute,University of Cape Town, Cape Town, South Africa; 5 Department of Family Medicine, University of California at Los Angeles, Los Angeles, California, United States of America; 6 Department of Psychiatry and Biobehavioral Sciences, University of California at Los Angeles, Los Angeles, California, United States of America; 7 Department of Molecular and Medical Pharmacology, University of California at Los Angeles, Los Angeles, California, United States of America; 8 The Brain Research Institute, University of California at Los Angeles, Los Angeles, California, United States of America

**Keywords:** Executive function, Methamphetamine use disorder, Contingency management

## Abstract

**Objectives:**

Contingency management is a promising intervention for Methamphetamine Use Disorder (MUD).Impaired executive function may decrease adherence to such treatment, but there are few data on whether impairment in executive function predicts treatment outcomes. We therefore evaluated whether baseline performance on tests of executive function predicted treatment response in a trial of contingency management for MUD.

**Methods:**

Thirty participants with MUD and 23 healthy controls performed the Connors Continuous Performance Task (CPT) and the Trail Making Task. MUD participants then entered an 8-week contingency management trial. Participants were categorized as responders (n=17; no methamphetamine-positive urine tests) or non-responders (n=13; >1 positive test). The Kruskal-Wallis test was used to compare scores in participants with MUD and healthy controls, and in responders versus non-responders.

**Results:**

Participants withMUD performed worse than controls on the CPT (d-prime) (p=0.012); non-responders performed worse than responders (p = 0.034). Performance of MUD participants did not differ significantly from controls on the Trail Making Task B (time to completion), but variation was high with non-responders performing worse than responders (p=0.013).

**Conclusion:**

These findings suggest that tests of executive function at baseline may be useful in predicting treatment response in MUD. Future work in larger samples may ultimately allow a more personalized treatment approach to methamphetamine use disorder.

## INTRODUCTION

Contingency management has shown promise in treating Methamphetamine Use Disorder (MUD) [1]. This treatment approach relies on the use of rewards for drug abstinence [2]. Notably, individuals with MUD exhibit compromised executive dysfunction [3-5], and such cognitive deficits have been linked to lower adherence to behavioural treatment [6]. In work by this group, scores on a test of decision-making, balancing rewards and penalties, participants who had a worse response to treatment also performed more poorly than those that did respond to treatment and healthy controls at baseline [7].

There are, however, few data on whether impairment in executive function predicts treatment response in MUD. Identifying predictors of treatment outcome can facilitate the development of personalized approaches to management. Yet the availability of individualized treatment for stimulant use disorders remains aspirational.

We recently demonstrated the efficacy of contingency management for MUD in a South African sample [2]. Here we employ these data to test the hypothesis that impairment in executive function at baseline predicts subsequent response to contingency management.

## METHODS

### Study Design

Data are from a pilot study evaluating an 8-week, escalating schedule of contingency management for treatment of MUD in a South African context. Full details of this trial are presented elsewhere [2]. The study was conducted according to the Principles of the Declaration of Helsinki; all research was overseen by the Health Science Human Research Ethics Committee of the University of Cape Town and the UCLA Institutional Review Board, and all participants provided written informed consent. Before participants entered the trial they completed the Wechsler Abbreviated Scale of Intelligence (WASI) [8] and the Montreal Cognitive Assessment (MoCA) [9] to evaluate overall cognitive function, as well as the Revised Hamilton Rating Scale for Depression (RHRSD) [10], the Addiction Severity Index (ASI) [11] and the Childhood Trauma Questionnaire [12]. Two laboratory tests of executive function were administered: the Connors Continuous Performance Task (CPT) [13] and the Trail-Making Task-B (TMT-B) [14].

### Research Participants

Potential participants who were not receiving treatment were recruited through advertisements, and others who were referred from treatment centres were receiving motivational interviewing as therapy. They were screened using the Structured Clinical Interview for DSM-5 (SCID-5) to identify those who met criteria for MUD. No other psychiatric comorbidities were allowed, except for Tobacco Use Disorder and Antisocial Personality Disorder, which are common co-morbidities associated with MUD [15-17]. Controls were matched using frequency matching to the MUD group on sex, race, age (age groups were as follows 18-22, 23-27, 28-32, 33-37, 38-42, 43-45), education (number of years of education were as follows 4-7, 8-10, 10-12, 13+), IQ (IQ ranges were as follows 60-69, 70-79, 80-89, 90-99, 100-109, 110-119, 120-129), Fagerström score, number of cigarettes smoked daily (between 0-4, 5-10, 11-15, 16-20 and 20+) and household income (SES score 1 to 5).

### Cognitive Tests

Both controls and MUD participants completed the cognitive tests in a quiet room with few or no distractions. Participants in the MUD group were tested before they entered the treatment trial, and control participants completed a baseline test session. The CPT was presented on a Dell Intel core i3 laptop, Vostro 2520 with a 15-inch screen using E-Prime software version 2.0., and the TMT-B was administered using paper and pencil.

The CPT and TMT-B were selected to evaluate different aspects of executive function. The CPT measures sustained attention, inattentiveness, impulsivity, and vigilance [18]. The primary outcome measure for this test was d-prime, which indicates the ability to discriminate targets from non-targets in response to cues. The TMT provides information on visual searching, scanning, speed of processing, and mental flexibility. Part B of the TMT test was used for this, and speed to completion was the primary outcome measure.

### Data Analysis

For each outcome measure (d-prime and time to completion), we determined whether the data were normally distributed, and then tested for homoscedasticity [19]. Since the data did not meet assumptions of normality and homoscedasticity, the non-parametric Kruskal Wallis Test was used to compare groups, and alpha was set at p<0.05. The Benjamini Hochberg adjustment was used to control for multiple comparisons with a false discovery rate of 0.05. In addition to comparing outcome measures in treatment responders vs non-responders, we also assessed whether MUD participants vs healthy controls was .associated with differences in executive function.

## RESULTS

Sociodemographic and clinical variables of research participants were tabulated (Table 1). The groups differed in education with controls having completed more years than the MUD group (p=0.009). Seven of the 30 participants in the MUD group but none of the controls met the diagnostic criteria for Antisocial Personality Disorder (p=0.001). The MUD group also was significantly more depressed than the controls (p=0.001).

With respect to the cognitive tasks (Table 2), responders to treatment had significantly greater d-prime than non-responders on the CPT (p=0.034), and also exhibited a significantly shorter time to completion on the TMT-B than non-responders (p=0.013). MUD participants had significantly lower d-prime than controls (p=0.012 on the CPT, but the groups did not differ in performance on the TMT-B p>0.05).

## DISCUSSION

Our findings confirm the hypothesis that treatment non-responders had worse executive function than responders, with responders having greater d-prime and having shorter time to completion on the CPT. This is a novel finding, and suggests that stronger attentional resources may enable patients to adhere to behavioural interventions over the short term. Such resources may correspond with capacity to engage escalating reinforcement procedures during contingency management to produce methamphetamine abstinence.

A comparison group of healthy controls, similar to the MUD group along most demographic and cognitive variables, completed more years of education than participants with MUD but scored similarly along a global measure of intellectual functioning that has been used for this purpose in South Africa [20]. This likely indicates that participants with MUD had early histories of social and educational disadvantage compared to controls. Other group differences highlighted factors common to persons diagnosed with MUD, including comorbid Antisocial Personality Disorder (Conduct Disorder as a child/adolescent) and elevated depression symptoms Neither of these factors that distinguished MUD participants from controls, however, interfered with treatment outcomes for MUD in U.S. [21,22].

A number of limitations deserve emphasis. First, the sample size is small. There is the potential for false negative findings, and we were unable to explore the impact of confounders such as comorbid depression on task performance. Second the scope of testing was limited. Aspects of executive function that should be explored in further work in MUD patients include suppressing an automated response as assessed by tasks such as the Hayling Sentence Completion [23], and discovery of rules, as measured using tasks such as the Brixton Spatial Anticipation Test [24].

Despite these limitations, we were able to confirm our hypothesis of an association between impaired executive function and treatment outcome in a trial of contingency management. Inclusion of executive function tests, such as the CPT, may be a useful part of an assessment of individuals with MUD prior to CM. It is possible that individuals with poor performance would benefit from a combination therapy including cognitive training [25] or a treatment augmentation using medications that can help reduce methamphetamine use during treatment [26-28] .

## CONCLUSION

The finding that responders to treatment performed better on tasks of executive function suggests that tests of this neurocognitive domain may be useful in predicting treatment response in MUD. Future work in larger samples may ultimately allow a more personalized treatment approach to methamphetamine use disorder.

## Figures and Tables

**Table 1:** Demographics.

	Means ± Std.dev			Means ± Std.dev		
	MUD group (n = 30)	Healthy Controls (n = 23)	Wilcoxon Rank Sum	Responders (n = 17)	Non-Responders (n = 13)	Wilcoxon Rank Sum
*Age*	34.3 ± 6.2	35.2 ± 7.0	p = 0.577	33.8 ± 6.7	35.1 ± 5.6	p = 0.706
*Race*	28 MRA, 2 African descent	21 MRA, 2 African descent	-	15 MRA, 2 African descent	13 MRA	-
*Education*	10.9 ± 2.9	12.5 ± 1.4	p = 0.009**	11.8 ± 2.9	9.6 ± 2.3	p = 0.055+
*WASI IQ*	84.7 ± 15.7	83.5 ± 15.8	p = 0.781	86.2 ± 18.4	82.9 ± 11.8	p = 0.706
*RHRSD*	26.6 ± 23.2	5.4 ± 6.1	p = 0.001****	28.7 ± 24.2	23.8 ± 22.5	p = 0.722
*Household Income (monthly)*	R16250.00 ± R15725.97	R 20108.70 ± R17113.89	p = 0.206	R9117.65 ± R11522.44	R25576.92 ± R15947.63	p = 0.009*
*Employment at time of trial*	9%	58%	-	16%	0%	-
*Cigarettes smoked daily*	8.3 ± 7.6	6.9 ± 6.7	p = 0.568	6.8 ± 6.0	10.2 ± 9.1	p = 0.486
*ASPD*	7	0	p = 0.001**	3	4	-
*ASI total drug score*				0.3 ± 0.1	0.3 ± 0.1	p = 0.690
*Grams per day*	1 ± 0.6	-	-	0.9 ± 0.5	1.1 ± 0.7	p = 0.372
*Years of misuse*	11.3 ±4.2	-	-	10.0 ± 4.4	12.9 ± 3.5	p = 0.119
*Amount spent monthly*	R1830.83 ± R1377.00	-	-	R1399.71 ± R1112.38	R2394.62 ± R1524.73	p = 0.062+
*Age initiated*	22.5 ± 6.3	-	-	22.4 ± 6.3	22.5 ± 6.5	p = 0.950
*Number of urine samples drug negative before scan*	3.8 ± 2.9	-	-	4.9 ± 3.3	2.3 ±1.0	p = 0.005**

Demographics data presented for between MUD and control groups, and within MUD group (ASI = Addiction Severity Index, ASPD = antisocial personality disorder, CTQ - Childhood trauma questionnaire, MRA = Mixed race ancestry, RHRSH = Revised Hamilton Rating Scale for Depression, stars (*) flag levels of significance with one star denoting a p value below 0.05, two if the p value is less than 0.01 and three for less that p = 0.001)

**Table 2:** Performance on Executive Function Tests.

Connors Continuous Performance Task D’prime	Median	Q1	Q3	IQR	Kruskal Wallis	Benjamini Hochberg
Heathy control	3.196	3.015	3.319	0.475	p = 0.012*	p = 0.012*
Methamphetamine Use Disorder	2.983	2.711	3.186	0.304		
Responders to treatment	3.013	2.823	3.21	0.387	p = 0.034*	p = 0.061+
Non-responders to treatment	2.937	2.69	3.052	0.362		
Trail Making Task B	Median	Q1	Q3	IQR	Kruskal Wallis	Benjamini Hochberg
Time to Completion						
MA Group	152.80	113.68	231.00	117.32	p = 0.554	p = 0.554
Healthy controls	116.50	89.355	214.7	125.345		
Responders to treatment	119.60	92.93	141.875	48.945	p = 0.013*	p = 0.039*
Non-responders to treatment	198.21	136.73	233.93	97.21		

Data are presented for 23 healthy control participants and 30 participants with Methamphetamine Use Disorder (17 responders to contingency management treatment, 13 non-responders). Data marked with an asterisk reached the criterion for statistical significance, the Benjamini Hochberg multiple comparisons correction was conducted for two tests.
